# Cutaneous Scalp Metastasis From Mammary Ductal Carcinoma

**DOI:** 10.7759/cureus.77187

**Published:** 2025-01-09

**Authors:** Michelle K Custer, Trevor Nessel, Joshua L Aron, Alexa F Israeli, Kevin T Nash, Edith Graves

**Affiliations:** 1 Dermatology, Edward Via College of Osteopathic Medicine, Auburn, USA; 2 Dermatology, Corewell Health, Farmington Hills, USA; 3 Dermatology, Lake Granbury Medical Center, Granbury, USA; 4 Dermatology, Nash Dermatology, Auburn, USA; 5 Oncology, East Alabama Medical Center, Opelika, USA

**Keywords:** breast cancer metastasis, cutaneous metastasis, dermatological manifestations, mammary ductal carcinoma, scalp involvement

## Abstract

The clinical progression of metastatic breast cancer may be indicated by dermatologic manifestations of the internal malignancy. Although uncommon, underlying malignancies can metastasize to the scalp and resemble various common dermatological lesions. We report the case of a 62-year-old female patient with a history of mammary ductal carcinoma who presented with a metastatic papule on the posterior mid-parietal scalp. Given that the cutaneous expression of internal malignancies is rare, it is imperative for dermatologists to conduct comprehensive patient histories and engage in multidisciplinary collaboration to optimize treatment strategies and improve patient prognosis.

## Introduction

The dermatologic manifestation of a patient’s internal malignancy is a unique finding as cutaneous metastases of primary malignancies constitute only 2% of all dermatologic malignancies [1]. More specifically, the scalp accounts for 2% of cutaneous malignant neoplasms [2]. Primary tumors located in the gastrointestinal tract, lungs, prostate, and breast are more commonly associated with scalp metastases [2]. Upon review of the English-language literature using PubMed-indexed data, fewer than 50 cases of scalp metastasis due to an underlying breast malignancy have been reported [2].

Although cutaneous manifestations of breast cancer may be present at initial diagnosis, it is more common for dermatologic lesions to present after diagnosis and treatment of the systemic malignancy [3]. The common areas of metastasis of breast cancer include the bone, lung, liver, and brain [4]. In patients with breast carcinoma, 23.9% presented with a dermatologic expression of their underlying malignancy [5]. The chest wall and abdominal area appear to be more common sites for the cutaneous manifestations of breast carcinoma, while the head, neck, and extremities are rarer sites [5].

Scalp metastasis from mammary ductal carcinoma presents with unique complications for diagnosis and prognosis. Cutaneous scalp metastases can visually mimic common dermatologic lesions such as basal cell carcinoma, squamous cell carcinoma, and sebaceous hyperplasia. The uncommon finding of scalp metastasis can be due to the vascular and lymphatic patterns in the scalp, which provide fewer routes for metastatic spread [6]. Understanding this rare metastatic pattern is necessary, as it indicates advanced diseases with poorer prognoses.

## Case presentation

**Figure 1:**
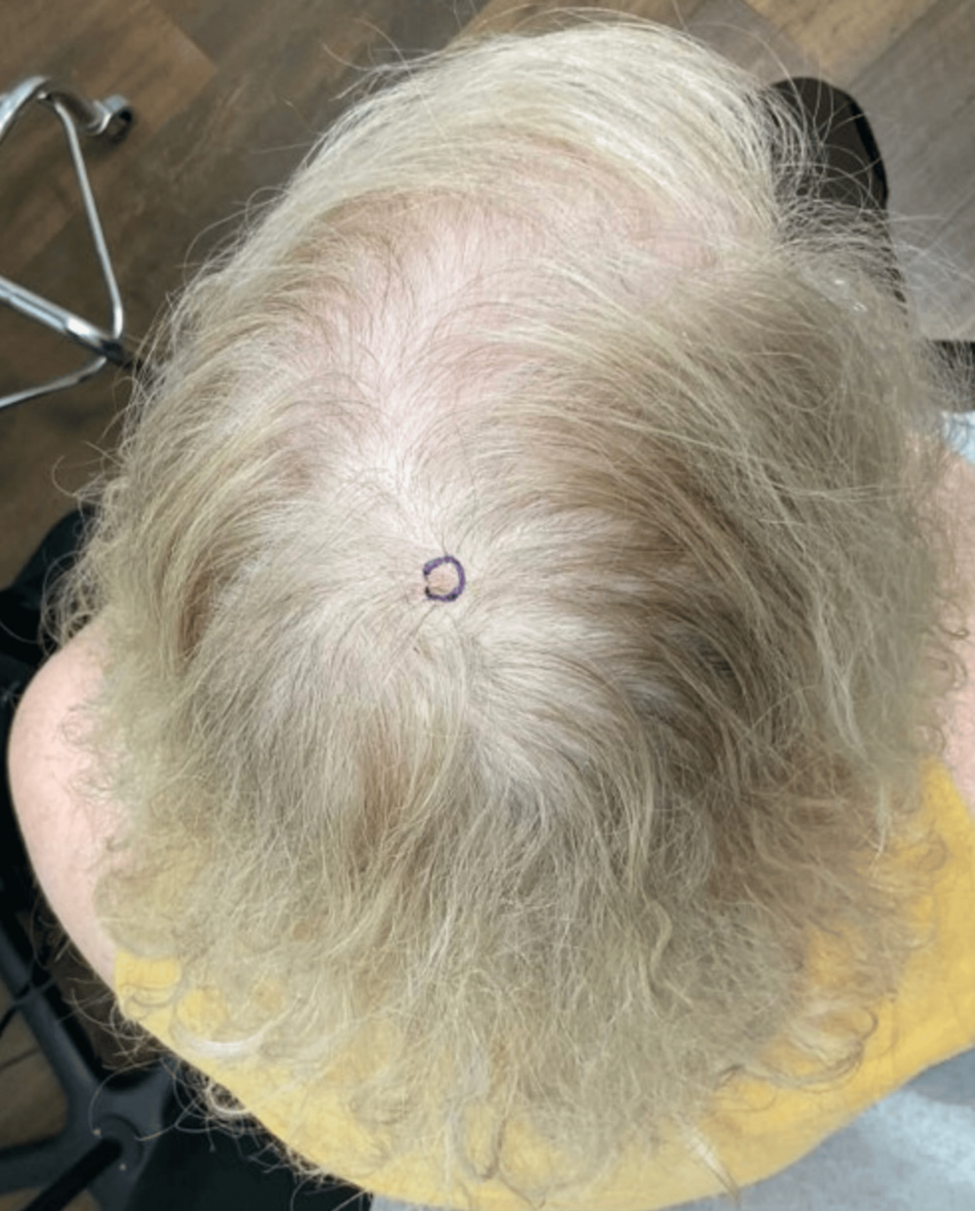
Initial Presentation of Cutaneous Scalp Lesion Solitary, well-circumscribed, pink, pearly, telangiectatic papule located on the posterior mid-parietal scalp.

A 62-year-old female patient presented to the outpatient dermatology clinic for the evaluation of a slightly erythematous, pearly, telangiectatic papule located on the posterior mid-parietal scalp (Figure 1). She has a family history of nonmelanoma skin cancer, but no personal history of previous skin cancer. Based on the appearance and location of the patient’s skin lesion, basal cell carcinoma was suspected, and a shave biopsy to the level of the superficial reticular dermis was performed.

The biopsy revealed an unremarkable epidermis, but the papillary dermis showed solar elastosis. Within the superficial reticular dermis and focally within the papillary dermis, irregular glandular units composed of atypical basaloid cells appeared to stem from the deeper, unsampled dermis. Additionally, small ductal acini were present. Atypical cells showed an increased nuclear-to-cytoplasm ratio and irregular nuclear membranes with prominent nucleoli.

In view of these findings, immunohistochemistry was performed, which was positive for cytokeratin 7 (CK7), estrogen receptor (ER), and GATA-3. The cells were nonreactive with cytokeratin 5/6 (CK5/6), p40, thyroid transcription factor-1 (TTF-1), Ber-EP4, and Melan-A. This immunoprofile is consistent with metastasis of mammary ductal carcinoma.

Further review of the patient’s medical history revealed that she had a history of multifocal upper outer quadrant right breast carcinoma and underwent bilateral mastectomies in 2012, followed by four cycles of docetaxel/cyclophosphamide completed in 2012, as well. She transitioned to letrozole, which she has continued to the present in addition to palbociclib for treatment of her metastatic disease.

Additionally, due to the patient’s development of a chronic cough, a computed tomography (CT) of the chest was ordered in 2023, which revealed dense consolidations, a segment of the lower lobe with obstruction of the segmental bronchus, and multiple sclerotic bone metastases in the ribs and several thoracic vertebral bodies. Positron emission tomography CT demonstrated widespread evidence of metastatic disease noted in the mediastinum, left hilum, liver, and numerous bony metastases. Due to the metastatic spread to the chest wall and skin, the patient was diagnosed with stage IV mammary ductal carcinoma.

**Figure 2:**
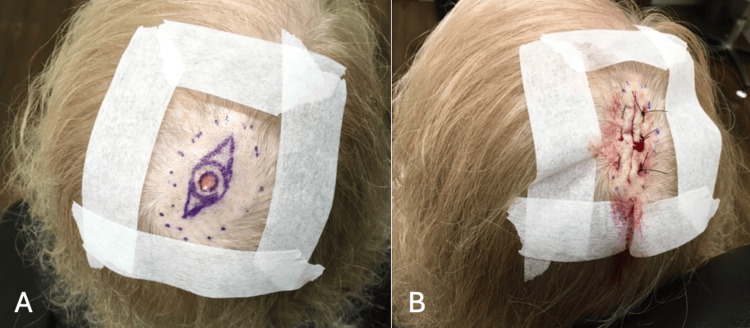
Preoperative and Postoperative Images of the Scalp Lesion (A) Scalp lesion prior to fusiform excisional procedure. (B) Fusiform excision closed by primary intention. The final wound length is 3.8 cm.

Following a discussion of the pathology results of the scalp lesion with the patient and consultation with the patient’s oncologist, the patient underwent a fusiform excision of the lesion (Figure 2). The surgical pathology report documented 0.10 mm perineural invasion and surgical excision margins free of carcinoma.

## Discussion

Cutaneous metastases vary in clinical appearance and presentation [7]. Although the most common presentation of cutaneous metastasis is in the form of nodules, other forms include dermatitis-like eruptions and inflammatory skin reactions [8]. Examples of dermatologic manifestations of an underlying breast malignancy include dermatomyositis, hypertrichosis lanuginosa acquisita, and erythema gyratum repens [9]. Biopsy and histopathological examination are needed for a definitive diagnosis.

The rarity of scalp metastasis can be attributed to the hematogenous and lymphatic spread of internal malignancies [6]. It is more common for cancer spread to occur through lymphatic channels, with primary neoplasms having a preferred cutaneous site of metastatic spread [10]. In patients with breast carcinoma, it is unusual for the primary site malignancy to spread to the head and neck region [5]. Hematological spread is due to cancer cells detaching from the primary neoplasm, traveling through blood vessels, and establishing growth in distant organs [10].

While metastatic breast cancer is associated with a poor prognosis, treatment of the systemic disease is aimed at symptomatic improvement, limitation of tumor progression, and prevention of recurrence [11]. Additionally, with a broad range of sites of metastasis, breast cancer treatment is tailored to the specific organs affected and prior treatment responses [11]. Treatment modalities for metastatic breast cancer include chemotherapy, endocrine therapy, radiation therapy, targeted therapy, and surgical intervention [12].

In this case, the patient was initially being treated with palbociclib with letrozole. However, analysis of the patient’s circulating tumor DNA revealed an alteration in estrogen receptor 1 (ESR1) and the progression of her disease despite therapy, which prompted the initiation of palliative treatment with elacestrant. Palliative radiation therapy to the upper lumbar spine was also administered, which provided symptomatic improvement. However, due to a lack of positive response after several weeks of treatment, elacestrant was discontinued and capecitabine treatment was initiated due to its favorable safety profile and use in treatment-resistant disease [13].

Additionally, it is crucial to note that the patient’s scalp metastasis displayed perineural invasion, which indicates a poor prognosis [14]. Perineural invasion implies disease infiltration along nerve sheaths, which may suggest a greater likelihood of local recurrence despite complete surgical excisions [14]. Notably, combined modality therapy is ideal for some patients with advanced disease as it can target multiple aspects of systemic disease, providing optimal symptomatic management and potentially increasing the survival rate [12].

## Conclusions

Cutaneous metastases of underlying internal malignancies can resemble the appearance of common dermatologic malignancies. Possessing knowledge of the rare occurrence of scalp metastases as a potential indicator of an underlying malignancy is essential, as it aids in prompt diagnosis, treatment, and improved outcomes. As a dermatologic lesion may indicate widespread disease, obtaining thorough patient medical histories and engaging in interdisciplinary collaboration are essential to developing optimal systemic treatment strategies and delivering comprehensive patient care.
